# Rabies vaccination strategies in the Netherlands in 2018: a cost evaluation

**DOI:** 10.2807/1560-7917.ES.2020.25.38.1900716

**Published:** 2020-09-24

**Authors:** Anita WM Suijkerbuijk, Marie-Josee J Mangen, Manon R Haverkate, Floriana S Luppino, Sabine E Bantjes, Leo G Visser, Corien M Swaan, Wilhelmina LM Ruijs, Eelco AB Over

**Affiliations:** 1 National Institute for Public Health and the Environment, RIVM, Bilthoven, the Netherlands; 2 These authors contributed equally to this manuscript; 3 Eurocross Assistance, Leiden, the Netherlands; 4 Leiden University Medical Center, Leiden, the Netherlands; 5 Department of Infectious Diseases, Leiden University, Leiden, the Netherlands

**Keywords:** costs, cost analysis, cost-effectiveness analysis, rabies, vaccination, post-exposure prophylaxis, pre-exposure prophylaxis, Netherlands

## Abstract

**Background:**

The risk of contracting rabies is low for travellers. However, the number of Dutch travellers potentially exposed abroad following an animal-associated injury and needing post-exposure prophylaxis (PEP) has increased, resulting in increased costs.

**Aim:**

Here, we evaluated the costs and the cost-effectiveness of different pre- and post-exposure interventions in the Netherlands, taking into account the 2018 World Health Organization (WHO) recommendations for the prevention of rabies.

**Methods:**

A decision tree-based economic model was constructed. We calculated and compared the cost of different WHO pre-exposure prophylaxis (PrEP) recommendations, intramuscular vs intradermal vaccination and PEP subsequent to increased vaccination coverage in risk groups. We estimated cost-effectiveness, expressed as incremental costs per rabies immunoglobulin (RIG) administration averted, using a societal perspective. Statistical uncertainty regarding number of travellers and vaccination coverage was assessed.

**Results:**

Total costs at the national level were highest using previous WHO recommendations from 2012, estimated at EUR 15.4 million annually. Intradermal vaccinations in combination with the current recommendations led to the lowest costs, estimated at EUR 10.3 million. Higher vaccination uptake resulted in higher overall costs. The incremental costs per RIG administration averted varied from EUR 21,300-46,800.

**Conclusions:**

The change in rabies PrEP and PEP recommendations in 2018 reduced total costs. Strategies with increased pre-travel vaccination uptake led to fewer RIG administrations and fewer vaccinations after exposure but also to higher total costs. Although larger scale intradermal administration of rabies vaccine can reduce total costs of PrEP and can positively influence vaccination uptake, it remains a costly intervention.

## Introduction

Rabies is a preventable infectious zoonotic disease that is responsible for annually roughly 59,000 deaths worldwide [1]. The majority of human rabies cases result from dog bites and following the onset of clinical symptoms, the disease is almost always fatal [2]. While rabies control heavily depends on prevention of rabies in dogs, vaccination of humans is an effective preventive intervention, either before or after exposure to the rabies virus [3,4]. Rabies vaccines are highly effective, safe and well tolerated [5]. For most travellers, the risk of contracting rabies is very low [6,7]. If organised access to medical care is available while travelling, including access to immediate care and rabies immunoglobulin (RIG), travellers may choose not to have pre-exposure vaccination (pre-exposure prophylaxis (PrEP)) [3]. Pre-travel immunisation is expensive and often not covered by health insurance.

An increasing number of Dutch travellers are potentially exposed to rabies virus abroad after an animal-associated incident (AAI) and consult a doctor for post-exposure prophylaxis (PEP) either abroad or after returning home. The National Institute for Public Health and the Environment (RIVM) receives on a daily basis requests for consultations by municipal health services, general practitioners, hospital specialists and medical repatriation organisations about potential rabies exposure accidents among Dutch travellers abroad or after returning home. The number of consultations increased from 184 in 2008 to 350 in 2014 and 450 in 2018 [8]. Consequently, the overall costs for seeking and receiving medical treatment after an AAI has increased.

According to the World Health Organization (WHO) classification, an AAI with potential exposure to rabies virus is defined as a category II or category III injury. A category II injury consists of nibbling of uncovered skin, minor scratches or abrasions without bleeding, while a category III consists of transdermal bites or scratches, contamination of mucous membrane or broken skin with saliva from animal licks. Depending on the category of exposure, PEP consists of extensive wound washing, administration of a series of rabies vaccines (RV) and RIG [2]. The RV administration varies in time-schedule (Essen vs Zagreb regimen) and injection technique (intradermal (ID) vs intramuscular (IM)). In this study, we considered the IM RV administration of 1 mL vaccine according to the Essen regimen, as the gold standard because that is the recommended regimen in the Netherlands. In the Netherlands, two vaccines are available: Rabipur produced by Glaxo Smith Kline (Marburg, Germany) and Rabies Mérieux produced by Sanofi Pasteur (Lyon, France). In case of a PrEP-naïve individual, not previously immunised but otherwise healthy, both exposure categories need the administration of RV. In case of a category III exposure, RIG is needed in addition. In case PrEP was previously given, only a short series of RV needs to be administered (Table 1). RIG is an expensive and globally scarce product that ideally should be used for those most at risk, which is the local population in endemic countries. Furthermore, an AAI needs a careful and often time-consuming assessment. These assessments frequently take place in stressful circumstances, as PEP needs to be started as soon as possible, preferably within 24 h after the AAI [9].

**Table 1:** Post-exposure prophylaxis scheme for rabies, the Netherlands, 2018

Category of exposure	PrEP	PEP
Category I (intact skin)	None	None
Category II (minor scratches or abrasions without bleeding)	Yes	2 × RV (day 0, 3)
	No	4 × RV (day 0, 3, 7, 14–28)
Category III (broken skin; contamination of mucous membrane with saliva)	Yes	2 × RV (day 0, 3)
	No	4 × RV (day 0, 3, 7, 14–28) + RIG (day 0)

PEP: post-exposure prophylaxis; PrEP: pre-exposure prophylaxis; RIG: rabies immunoglobulin; RV: rabies vaccination.

Source: [9,10].

The current WHO recommendations on PEP treatment were presented in 2018 and differed from the previous recommendations mainly on two points: Firstly, the number of needed vaccination doses was lowered by one dose for both PrEP and PEP. Secondly, the dose of RIG was based on the anatomical localisation of the affected area instead of the body weight of the individual [10]. Maximum infiltration of RIG into and around the wound is effective but the benefit from additional IM administration of any remaining RIG based on a person’s body weight at a site distant to the wound may be limited [2,11,12]. The amount of administered RIG is therefore in almost all cases based on the size of the wound. Only the maximum dose of RIG is still assessed by body weight. Since the introduction of the current recommendations, the amount of RIG is estimated to be on average 40% of the quantity that was previously required based on body weight [9,10]. Hence, the 2018 WHO rabies recommendations are expected to have a positive effect on the costs of rabies prevention for the individual traveller and at national level. A schematic overview of the main differences between the 2018 guidelines and those in place before 2018 is given in Table 2.

**Table 2:** Rabies vaccination dosage in current and former recommendations, the Netherlands, 2018

Rabies vaccination	Current recommendations	Former recommendations
PrEP	2 doses	3 doses
PEP (not previously immunised)	4 doses	5 doses
PEP (previously immunised people)	2 doses	2 doses
RIG	2–10 mL with a maximum based on body weight	20/40 IU/kg

IU: international units; PEP: post-exposure prophylaxis; PrEP: pre-exposure prophylaxis; RIG: rabies immunoglobulin.

In the WHO recommendations, rabies vaccinations can be administered either IM or ID. One ID dose is 0.1 mL of vaccine, leading to an immune response comparable with IM administration but at a considerably lower dose, thus saving vaccine and costs [9,13]. A disadvantage is that vaccination staff must be well trained to guarantee full ID instillation of the vaccine and to avoid accidental subcutaneous injection. In the Netherlands, ID administration is used off-label and only practiced for PrEP in a few vaccination centres; a two-site ID vaccination is given twice. Therefore, in this study, ID administration of the vaccine is only considered for PrEP rather than as outlined in the former or current recommendations.

The current WHO recommendations for rabies PrEP and PEP, as implemented in the Netherlands, are expected to have a positive effect on the costs of rabies prevention for the individual traveller and at national level. The ID vaccination can also contribute to cost reduction. In the Netherlands, several studies have focused on clinical outcomes or on the effect of risk behaviour of Dutch travellers on the number of AAI [14,15]. However, to our knowledge, an economic study investigating the possible cost reduction of different rabies prevention approaches is lacking. This study aims to assess the costs and the cost-effectiveness of different preventive interventions for rabies, by systematically comparing different strategies: (i) cost-effectiveness reached with the implementation of the 2018 WHO recommendations, compared with the recommendations before 2018, (ii) costs of ID vs IM vaccination and (iii) costs-effectiveness of an increase in PrEP in Dutch travellers in order to avoid extensive PEP regimens.

## Methods

We investigated costs of several policy measures for rabies in the Netherlands using a decision tree-based economic model programmed in MS Excel 2010. With this model, costs of different prevention strategies, including their effect on subsequent post-exposure treatment can be calculated and compared per risk group (see chapter *Assessment of risk groups*), both at national level and per person vaccinated before travelling. Two time points were included in the model: before travelling at the travel clinic when a risk assessment is made, and after an AAI when PEP is considered.

Firstly, we assessed the economic impact of the 2018 PrEP recommendations vs former recommendations, both with IM administration of the vaccine. Secondly, we compared ID vs IM administration of PrEP (two-dose administrations). Thirdly, we analysed the effects of a 1.5-fold increase in the uptake of PrEP in risk groups, using IM administration (two applications). We assumed that the increased vaccination uptake would happen because of revised recommendations and only within the traveller population who obtained a medical consultation at Municipal Health Services (MHS), general practitioners (GPs) or another travel clinic before their journey. No additional campaigns to increase the number of travellers requesting a medical consultation before their journey were assumed, hence no additional campaign costs for the MHS and GPs were included.

PEP with vaccinations is indicated for category II exposures, according to WHO recommendations (Table 1). PEP is indicated with vaccine and, for those without PrEP, with administration of RIG for category III exposures (Table 1). We estimated cost differences between the former and current vaccination policies following recommendations of WHO including differences in the amount of RIG. In addition, we calculated cost implications when implementing ID administration of rabies vaccine using the current vaccination policy with two doses as comparator strategy. We present costs of PrEP for several risk groups of travellers (see chapter *Assessment of risk groups*), both at national level and per vaccinated person. The benefit of higher pre-travel vaccination coverage among risk groups is the reduced use of RIG and a less extensive vaccination scheme after possible exposure. We calculated the incremental costs per (extra) vaccinated person and incremental costs per RIG application averted as an indication of the cost-effectiveness of the prevention strategy. A time horizon of 1 year was considered. The economic model was employed from a societal perspective, including healthcare costs, costs of lost holidays and an impact on evacuation and repatriation of those travellers possibly exposed, costs that would have to be covered by the patients themselves, their travel insurance and/or their health insurance.

### Assessment of risk groups

In 2017, a case–control study was conducted among Dutch travellers to rabies-endemic countries in which the various determinants of possible exposure to rabies virus were investigated [14]. The following risk groups were identified that would be eligible for more intensive rabies prevention: (i) men, (ii) travellers younger than 35 years and (iii) travellers to south-eastern and western Asia as these groups had a higher risk of a rabies-associated incident during travel. In addition, we added (iv) travellers to South America to this list as in South America little or no RIG is available for travellers in case of AAI so that repatriation or evacuation of unvaccinated patients with a category III wound is required [16].

### Pre-travel consultations

In the Netherlands, the decision whether rabies vaccinations are indicated before and/or after an AAI and the actual administration of the vaccinations are provided by MHS, GP services, some hospital’s outpatient clinics and some commercial vaccination centres. The decision-making is based on travel destinations, travel duration, accommodations and activities during travel and on personal conditions including vaccination history. For our model, we used data from travellers consulting the MHS Hart voor Brabant and MHS Amsterdam between 1 July 2016 and 1 July 2017 (for details see Supplement).

### Travellers needing post-exposure prophylaxis

The estimated annual number of people who presented to a healthcare provider because of an AAI while travelling is provided in Table 3. The number of RIG administrations that were actually indicated, and provided once returned to the Netherlands, was based on the registration of RIG therapies by the RIVM in 2017. For the number of administered RIG abroad while travelling, figures from Eurocross Assistance (ECA) were used (see Supplement). We extrapolated the mean number of RIG administrations abroad (n = 64) in the ECA database between 2016 and 2018 to all travellers needing RIG abroad, taking into account the market share of ECA (30%, total n = 213). For incidents where RIG was not indicated, registration data from all Dutch MHS were used. ECA also provided the number of incidents requiring only two vaccinations abroad (for travellers with PrEP). This figure was also extrapolated to the Netherlands. To prevent double counting, travellers from the ECA registry needing PEP who started their regimen abroad with a full series of four vaccinations but further completed their vaccinations at MHS in the Netherlands were not taken into account.

**Table 3:** Annual number of people possibly exposed to rabies virus, the Netherlands, 2016–2018 (n = 1,400)

Type of PEP	n	PrEP	Percentages
Category III injury needing RIG and full series of vaccinations^a^	413	No	29.5%
Category III injury, no RIG indication^b^, full series of vaccinations^a^	540	No	38.6%
Category III injury needing two vaccinations	285	Yes	20.3%
Category II injury needing full series of vaccinations^a^	125	No	8.9%
Category II injury needing two vaccinations	37	Yes	2.7%
**Total**	**1,400**	**NA**	**100%**

NA: not applicable; PEP: post-exposure prophylaxis; PrEP: pre-exposure prophylaxis; RIG: rabies immunoglobulin.

^a^ Persons without PrEP, using current recommendations: four vaccinations; using former recommendations: five vaccinations.

^b^ >7 days after start of PEP vaccination series RIG is not indicated anymore and according to Dutch guidelines, RIG is not indicated for injuries inflicted by monkeys.

### Costs

Estimated costs were determined for both PrEP and PEP courses, including costs for the vaccine and RIG, administration costs, management costs for handling PEP, costs of lost holidays and costs involved in moving the involved subjects from one facility to another in case the required treatment needed was not available (Table 4). For the administration of RIG some cases needed repatriation to the Netherlands, other cases needed evacuation to another country abroad, for example to a neighbouring country. Hospitalisation costs were not included as an AAI generally does not lead to further treatment and care in a hospital. All costs were indexed to Euros 2017 using Dutch consumer price indexes (www.cbs.nl). Detailed information about costs is provided in the Supplement.

**Table 4:** Costs for rabies PrEP and PEP in Euros, the Netherlands

Costs before travelling	MHS cost in EUR	GP cost in EUR	Source
First consultation (per person)	13.10	67.56	Own calculations based on survey^a^
Follow up consultation (per person)	5.45	16.89	Own calculations based on survey^a^
Vaccine costs per IM administration	51.64	51.64	[31]
Vaccine costs per ID administration	6.81^b^	NA	Own calculations based on survey^a^
Delivery fee for pharmacy (first time)	NA	14	[32]
Delivery fee for pharmacy (second time and further)	NA	7	[32]
Vaccination card	4.09	4	Mean price found on travel clinic websites [33,34]
**Costs after animal-associated incident**	**Costs**	**Source**	
Management MHS for persons needing RIG	276	Own calculations based on survey^a^	
Management RIVM for persons needing RIG	371	Own calculations based on survey^a^	
Transportation RIG	125	Mean costs, retrieved from RIVM	
RIG old recommendations (new recommendations), administered in the Netherlands	2,000 (800)	Own calculations (2 mL = EUR 375) (smaller dose)	
RIG, administered abroad	2,731	Mean costs per case, ECA	
Administration RIG	31.76	Own calculations based on survey^a^	
Management MHS for persons not needing RIG	98.64	Own calculations based on survey^a^	
Vaccine	51.64	[31] per dose	
Administration vaccination	7.63	Own calculations based on survey^a^	
Mean repatriation costs	1,650	Costs per case, ECA^c^	
Mean evacuation costs	1,844	Costs per case, ECA^d^	

ECA: Eurocross Assistance; GP: general practitioner; ID: intradermal administration; IM: intramuscular administration; MHS: Municipal Health Service; NA: not applicable; PEP: post-exposure prophylaxis; PrEP: pre-exposure prophylaxis; RIG: rabies immunoglobulin; RIVM: National Institute for Public Health and the Environment.

All costs are expressed in Euros 2017.

^a^ For details see Supplement (time costs of medical personnel including overhead expenses).

^b^ The two-site ID regimen is included, therefore four doses in total.

^c^ 9.5% of all persons needing RIG have repatriation costs.

^d^ 7.4% of all persons needing RIG have evacuation costs.

### Cost-effectiveness

We calculated the incremental cost-effectiveness ratio (ICER) per (extra) person vaccinated before travelling and the ICER per RIG administration averted as an indication of the cost-effectiveness of the prevention strategy. That means that we divided the difference in total costs by the difference in persons vaccinated before travelling and we divided the difference in total costs by the difference in RIG administrations.

### Sensitivity analysis

Statistical uncertainty with respect to the number of travellers to certain regions and to vaccination coverage estimates was considered using the Monte Carlo simulation technique and the add-in software Palisade @Risk 7.5 in which we sampled 10,000 random values from input distributions. All other variables were considered fixed by using average resource utilisation and fixed unit cost prices. Results are presented as means with 95% uncertainty intervals.

## Results

Table 5 shows the costs of PrEP and PEP according to the former and current recommendations, and if vaccinations would be administered ID. In all these situations, we considered the current vaccination uptake in combination with the existing number of rabies-associated incidents. The number of visitors to a vaccination centre before travel to a rabies-endemic country was 353,100, of whom 37,300 persons were vaccinated during the visit. Among vaccinated travellers, the number of AAI was 322. Among the 1,078 unvaccinated travellers having an AAI, 665 persons did not receive RIG because of (i) a category II injury, (ii) a category III injury where it was too late for the RIG administration for instance because RIG was not available (RIG has to be given within 7 days after start of PEP vaccination series) or (iii) a category III injury inflicted by monkeys. Among 953 unvaccinated travellers having an AAI with a category III injury, 413 persons received RIG. Costs were highest following the former recommendations, estimated at EUR 15.4 million annually for the Dutch society (EUR 411 per vaccinated person). Overall costs were lowest when ID vaccinations were administered, estimated at EUR 10.3 million per year (EUR 275 per vaccinated person). Of all persons needing RIG, 9.5% had repatriation costs and 7.4% had evacuation costs.

**Table 5:** Annual costs of rabies PrEP and PEP using different regimens, assuming no change in vaccination uptake, the Netherlands, 2018

	Former recommendations IM	Current recommendations IM	ID
National costs for prevention (before travel) in EUR			
Consultation	7,050,700	6,807,800	6,807,800
Vaccination	5,878,700	3,927,200	1,353,100
**Total (before travel)**	**12,929,400**	**10,735,000**	**8,160,900**
National costs for prevention after an AAI in EUR			
Vaccinations and RIG	1,401,300	1,086,400	1,086,400
Consultation and coordination	480,900	480,900	480,900
Repatriation	64,800	64,800	64,800
Evacuation	56,400	56,400	56,400
Lost holidays	423,000	423,000	423,000
**Total (after potential exposure) in EUR**	**2,426,400**	**2,111,500**	**2,111,500**
**Grand total (national costs) in EUR**	**15,355,800**	**12,846,500**	**10,272,400**
Costs in EUR per person vaccinated before travel	411	336	275

ID: intradermal administration; IM: intramuscular administration; PEP: post-exposure prophylaxis; PrEP: pre-exposure prophylaxis; RIG: rabies immunoglobulin.

Results of increasing the vaccination coverage with 150% in the identified risk groups are presented in Table 6 and Table S3 in the Supplement. The largest risk group were people younger than 35 years, followed by people travelling to south-eastern and western Asia. In all risk groups, a higher vaccination uptake led to higher overall costs, despite a decline in management and vaccination costs of possible rabies exposure incidents, and avoided annually between 4 and 60 cases requiring RIG. The costs per averted RIG administration varied from EUR 21,300 to EUR 46,800. The ICER per additional person vaccinated before travelling varied between EUR 98 and EUR 105 per additional vaccinated person. This is also illustrated in Supplementary Figures S1 and S2. 

**Table 6:** Costs, RIG administrations and ICER as a result of a 1.5-fold increased rabies vaccination uptake for diverse risk groups, the Netherlands, 2018

Risk group	Travellers < 35 years	Travellers to south-eastern and western Asia	Men	Travellers to South America	Travellers having at least one risk factor	Travellers having all risk factors together^a^
Additional vaccinated persons	11,970	9,800	8,570	1,800	17,290	3,410
95% UI	2,850–22,100	1,880–18,530	1,280–16,540	−3,280–6,730^b^	51,60–31,160	−1,750–8,810^b^
Avoided number of RIG administrations	45	41	29	4	60	16
Additional costs	1,206,000	966,400	867,100	188,900	1,755,100	333,100
95% UI	226,000–2,413,900	421,000–2,290,000	77.400–1,819,800	−391,900–765,300^b^	482,700–3,341,000	−260,600–941,800^b^
ICER: Incremental costs per additional vaccinated person	101	99	101	105	102	98
95% UI	82–116	71–116	0.75–118.0	71–151	88–116	26–181
ICER: Incremental costs per avoided RIG administration	26,970	23,760	30,370	46,750	29,220	21,320
95% UI	9,530–52,460	5,790–49,510	5,323–64,110	−129,950–229,830^b^	14,250–53,000	−24,010–70,190^b^

ICER: incremental cost-effectiveness ratio; RIG: rabies immunoglobulin; UI: uncertainty interval.

^a^ This group consist of men younger than 35 years travelling to south-eastern and western Asia.

^b^ Owing to low numbers in this specific risk group, the UI are not interpretable.

The table presents averages with 95% UI. Note that in Table 4 total costs per vaccinated persons are presented, whereas in Table 5 the incremental costs per additional vaccinated person are presented, and they are therefore not comparable. For full details see Supplementary Table S3.

## Discussion

This cost evaluation showed that the current WHO rabies PrEP and PEP recommendations, as implemented in 2018 in the Netherlands, were associated with a substantial reduction (16%) in societal costs. An additional cost reduction (33%) could be achieved by offering ID vaccination on a large(r) scale. Increased use of ID administration requires proper planning of the number of travellers who need a rabies vaccination in order to avoid wasting vaccine. Therefore, ID administration would not lead to vaccine savings if there are only a few cases. A hypothetical increased vaccination uptake in the defined risk groups would lead to higher overall costs, despite the smaller number of RIG administrations and a reduced number of vaccinations after an AAI. Even though the costs of RIG administration according to the new recommendations were lower (currently EUR 800 instead of the former EUR 2,000 in the Netherlands), total costs were higher because of higher costs of PrEP when assuming a higher number of travellers vaccinated before travelling. As the incidence of AAI in travellers to endemic countries is low, increasing the vaccination uptake is a costly intervention with an ICER varying from EUR 21,300 to EUR 46,800 per avoided RIG administration. These results are not only relevant for the Netherlands but also for other European countries with a similar healthcare system. The risks of contracting rabies are comparable for travellers from other countries and the costs of preventing rabies will also be similar.

Our results are in line with a previous modelling study by Hampson et al. [17]. In that study, WHO recommendations for use of human rabies vaccines were modelled to inform prophylaxis regimens to prevent human rabies. The ID vaccination regimen was recommended as it is less costly and treats more patients when vaccine is in short supply. In a cost evaluation of Le Guerrier et al. it was found that PrEP given routinely to Canadian travellers heading for rabies-endemic regions was far too costly and therefore not indicated [18], in contrast to people living in endemic countries, where routine PrEP vaccination programmes can be an efficient use of resources [19]. The Strategic Advisory Group of Experts on Immunization (SAGE) committee stated that PrEP, as a large scale public health intervention, is not cost-effective and would have acceptable costs only in areas where RIG is rarely administered and the dog bite incidence exceeds 6% [2]. PrEP can play a valuable role in protecting high-risk populations in remote areas, especially where the risk of bat rabies is not easily controlled [9]. Kessels et al. systematically reviewed cost-effectiveness of national programmes implementing PrEP for high-risk populations in remote settings [20].

This study has several strengths and limitations. One of the strong points was the use of model input data from different MPH and a recently conducted case–control study. The first limitation concerns the period of data collection, during which the old vaccination recommendations still applied. It is plausible that the vaccination uptake will go up as the number of necessary doses and consequently the costs and efforts for the traveller decrease. Secondly, because of data restrictions, the model contained a time horizon of only 1 year while vaccination offers lifelong immunological memory, thereby underestimating the benefits of improved vaccination coverage, which accumulates with every next trip to a rabies-endemic region. At present, decisions to vaccinate before travelling are typically made on the basis of an individual risk–benefit assessment and individual willingness to pay for the vaccine [21]. If future travel is incorporated into the considerations of risk exposure, the decision regarding vaccination might change. Finally, we did not include in our model costs for medical assistance organisations and costs for increasing the vaccination uptake among risk groups such as campaign costs, thereby underestimating total costs.

PrEP is indicated for individuals who face occupational and/or travel-related exposures to rabies virus in specific settings or over an extended period of time [9]. Many travellers are not aware of the health risks during their travel. Lammert et al. evaluated a large cohort of international travellers who obtained a pre-travel health consultation at clinics in the United States that provide health advice for international travellers [22]. They found that a large part (28%) of travellers who sought pre-travel health advice rejected some of the recommended vaccinations. A lack of concern about the associated illnesses was the most frequently mentioned reason to refuse the vaccines. More specifically, Marano et al. assessed rabies risk perception among individuals who travelled to rabies-endemic countries [23]. Within the subsample of travellers at higher risk for rabies, a large part (83%) was aware of the basic characteristics of rabies. However, only 8% reported receiving PrEP vaccination within the past 3 years. On addition, vaccination costs and a short time period until travel proved to be barriers for rabies vaccination [15]. Therefore, additional effective education about rabies risks seems necessary for high-risk groups.

Other preventive measures to be taken in endemic countries such as One Health approaches have proven (cost-)effective in controlling rabies in different areas of the world [24,25]. For example, an economic evaluation performed in rural India indicated that a combination of a dog vaccination campaign, sterilisation of free-roaming dogs and PEP in humans after dog bites is likely to provide the optimal scenario for cost-effective prevention of human rabies [26]. In Chad, besides mass dog vaccination, improved communication between human health and veterinary workers was imperative to prevent human rabies deaths through the appropriate use of PEP [27]. Also in Tanzania, integrated control programmes proved to be cost-effective [28]. In the first place, these measures contribute to protection of the local population and have also positive externalities (are beneficial) for (international) travellers.

There is some evidence that vaccination schemes can be further simplified, as Jonker and Visser found promising results that vaccinating with a single dose was already sufficient to induce an adequate anamnestic antibody response in all subjects, at least for 1 year [29]. However, a single PrEP vaccination is only viable if additional scientific evidence on efficacy of this reduced PrEP scheme becomes available. In Europe, rabies is a very rare infectious disease and it is almost always associated with travel to an endemic country [30]. It remains important to inform the public, especially those travelling to endemic areas, about the risk of contracting rabies and to consider PrEP for those with increased risk. This study revealed that rabies-associated incidents are a cause of concern during travel. It involves many resources and costs for local professionals and healthcare providers in the home country, often in tense situations. Increased uptake of PrEP in travellers is accompanied by higher cost. The ID administration of rabies vaccination can reduce total costs of PrEP, positively influencing the vaccination uptake.

## Supplementary Data

232000 Supplement

## References

[1] Hampson K Coudeville L Lembo T Sambo M Kieffer A Attlan M Estimating the global burden of endemic canine rabies. PLoS Negl Trop Dis. 2015;9(4):e0003709. 10.1371/journal.pntd.0003709 25881058 PMC4400070

[2] SAGE working group. Background paper: proposed revision of the policy on rabies vaccines and rabies immunoglobulins. Geneva: World Health Organization; 2017. Available from: https://www.who.int/immunization/sage/meetings/2017/october/1_Background_paper_WG_RABIES_final.pdf?ua=1

[3] Crowcroft NS Thampi N The prevention and management of rabies. BMJ. 2015;350(jan14 26):g7827. 10.1136/bmj.g7827 25589091

[4] Lankau EW Cohen NJ Jentes ES Adams LE Bell TR Blanton JD Prevention and control of rabies in an age of global travel: a review of travel- and trade-associated rabies events--United States, 1986-2012. Zoonoses Public Health. 2014;61(5):305-16. 10.1111/zph.12071 23870674

[5] World Health Organization Rabies vaccines: WHO position paper, April 2018 - Recommendations. Vaccine. 2018;36(37):5500-3. 10.1016/j.vaccine.2018.06.061 30107991

[6] Steffen R Behrens RH Hill DR Greenaway C Leder K Vaccine-preventable travel health risks: what is the evidence--what are the gaps? J Travel Med. 2015;22(1):1-12. 10.1111/jtm.12171 25378212

[7] Carrara P Parola P Brouqui P Gautret P Imported human rabies cases worldwide, 1990-2012. PLoS Negl Trop Dis. 2013;7(5):e2209. 10.1371/journal.pntd.0002209 23658853 PMC3642086

[8] Eurocross Assistance. Steeds meer reizigers maken melding van mogelijke besmetting met hondsdolheid. [More and more travellers report possible rabies infection]. Leiden: Eurocross Assistance; 2018. Dutch. Available from: https://www.eurocross.nl/nieuws/2018/steeds-meer-reizigers-maken-melding-van-mogelijke-besmetting-met-hondsdolheid/

[9] World Health Organization (WHO). WHO expert consultation on rabies: third report. Geneva: WHO; 2018. Available from: https://apps.who.int/iris/handle/10665/272364

[10] Rijksinstituut voor Volksgezondheid en Milieu (RIVM). Rabiës richtlijn. [Rabies guideline]. Bilthoven: RIVM. [Accessed: 18 Sep 2019]. Dutch. Available from: https://lci.rivm.nl/richtlijnen/rabies

[11] Bharti OK Madhusudana SN Gaunta PL Belludi AY Local infiltration of rabies immunoglobulins without systemic intramuscular administration: An alternative cost effective approach for passive immunization against rabies. Hum Vaccin Immunother. 2016;12(3):837-42. 10.1080/21645515.2015.1085142 26317441 PMC4964710

[12] Bharti OK Madhusudana SN Wilde H Injecting rabies immunoglobulin (RIG) into wounds only: A significant saving of lives and costly RIG. Hum Vaccin Immunother. 2017;13(4):762-5. 10.1080/21645515.2016.1255834 28277089 PMC5404375

[13] Kong LY Vincelette J Laplante G Duchesne JA Libman M Barkati S Intradermal pre-exposure rabies vaccination in a Canadian travel clinic: 6-year retrospective observational study. CMAJ Open. 2018;6(2):E168-75. 10.9778/cmajo.20170147 29636332 PMC7869659

[14] Bantjes S, Haverkate M, Ruijs H, van den Hoogen G, Croughs M, Pijtak A, et al. Predictors of possible rabies exposure in travelers: a case-control study. Eur J Public Health. 2018;28(suppl_4).

[15] Wieten RW Tawil S van Vugt M Goorhuis A Grobusch MP Risk of rabies exposure among travellers. Neth J Med. 2015;73(5):219-26. 26087801

[16] van Dee L, Dimmendaal M, Bantjes S, Haverkate M, van Kessel R. Rabiëscasuïstiek in de GGD-regio Utrecht. [Rabies in the MHS region Utrecht]. Infectieziekten Bulletin. 2018;29(9). Dutch. Available from: https://magazines.rivm.nl/2018/11/infectieziekten-bulletin/rabi%C3%ABscasu%C3%AFstiek-de-ggd-regio-utrecht-0

[17] Hampson K Abela-Ridder B Bharti O Knopf L Léchenne M Mindekem R Modelling to inform prophylaxis regimens to prevent human rabies. Vaccine. 2019;37(Suppl 1):A166-73. 10.1016/j.vaccine.2018.11.010 30528846 PMC7612382

[18] LeGuerrier P Pilon PA Deshaies D Allard R Pre-exposure rabies prophylaxis for the international traveller: a decision analysis. Vaccine. 1996;14(2):167-76. 10.1016/0264-410X(95)00110-M 8852415

[19] Anothaisintawee T Julienne Genuino A Thavorncharoensap M Youngkong S Rattanavipapong W Meeyai A Cost-effectiveness modelling studies of all preventive measures against rabies: A systematic review. Vaccine. 2019;37(Suppl 1):A146-53. 10.1016/j.vaccine.2018.11.071 30554795

[20] Kessels JA Recuenco S Navarro-Vela AM Deray R Vigilato M Ertl H Pre-exposure rabies prophylaxis: a systematic review. Bull World Health Organ. 2017;95(3):210-219C. 10.2471/BLT.16.173039 28250534 PMC5328107

[21] Leder K Chen LH Wilson ME Aggregate travel vs. single trip assessment: arguments for cumulative risk analysis. Vaccine. 2012;30(15):2600-4. 10.1016/j.vaccine.2011.12.133 22234265

[22] Lammert SM Rao SR Jentes ES Fairley JK Erskine S Walker AT Refusal of recommended travel-related vaccines among U.S. international travellers in Global TravEpiNet. J Travel Med. 2016;24(1):taw075. 10.1093/jtm/taw075 27799502 PMC5091771

[23] Marano C Moodley M Melander E De Moerlooze L Nothdurft HD Perceptions of rabies risk: a survey of travellers and travel clinics from Canada, Germany, Sweden and the UK. J Travel Med. 2019;26(Suppl 1):S3-9. 10.1093/jtm/tay062 30476212 PMC6377182

[24] Cleaveland S Lankester F Townsend S Lembo T Hampson K Rabies control and elimination: a test case for One Health. Vet Rec. 2014;175(8):188-93. 10.1136/vr.g4996 25172649 PMC7612423

[25] Rysava K Miranda ME Zapatos R Lapiz S Rances P Miranda LM On the path to rabies elimination: The need for risk assessments to improve administration of post-exposure prophylaxis. Vaccine. 2019;37(Suppl 1):A64-72. 10.1016/j.vaccine.2018.11.066 30573356 PMC6863041

[26] Fitzpatrick MC Shah HA Pandey A Bilinski AM Kakkar M Clark AD One Health approach to cost-effective rabies control in India. Proc Natl Acad Sci USA. 2016;113(51):14574-81. 10.1073/pnas.1604975113 27994161 PMC5187709

[27] Lechenne M Mindekem R Madjadinan S Oussiguéré A Moto DD Naissengar K The importance of a participatory and integrated one health approach for rabies control: the case of N’Djaména, Chad. Trop Med Infect Dis. 2017;2(3):43. 10.3390/tropicalmed2030043 30270900 PMC6082095

[28] Lankester F Davis A Kinung’hi S Yoder J Bunga C Alkara S An integrated health delivery platform, targeting soil-transmitted helminths (STH) and canine mediated human rabies, results in cost savings and increased breadth of treatment for STH in remote communities in Tanzania. BMC Public Health. 2019;19(1):1398. 10.1186/s12889-019-7737-6 31660915 PMC6819457

[29] Jonker EFF Visser LG Single visit rabies pre-exposure priming induces a robust anamnestic antibody response after simulated post-exposure vaccination: results of a dose-finding study. J Travel Med. 2017;24(5). 10.1093/jtm/tax033 28931127

[30] European Centre for Disease Prevention and Control (ECDC). Rabies – Annual epidemiological report for 2018. Stockholm: ECDC; 2019. Available from: https://www.ecdc.europa.eu/en/publications-data/rabies-annual-epidemiological-report-2018

[31] Zorginstituut Nederland. Rabiesvaccin merieux flacon 2,5IE+solvens 1ml. [Rabies vaccine merieux vial 2.5IE + solvens 1ML]. Diemen: Zorginstituut Nederland. [Accessed: 18 Sep 2019]. Dutch. Available from: https://www.medicijnkosten.nl/medicijn?artikel=RABIESVACCIN+MERIEUX+FLACON+2%2C5IE+%2B+SOLVENS+1ML&id=91558d973ee8763efce00192f24d314e

[32] Zorginstituut Nederland. Richtlijn voor het uitvoeren van economische evaluaties in de gezondheidszorg. [Guideline for conducting economic evaluations in healthcare]. Amsterdam: Zorginstituut Nederland; 2016. Dutch. Available from: https://www.zorginstituutnederland.nl/publicaties/publicatie/2016/02/29/richtlijn-voor-het-uitvoeren-van-economische-evaluaties-in-de-gezondheidszorg

[33] Gemeenschappelijke Gezondheidsdienst (GGD). Reisvaccinaties. Tarieven. [MHS tariffs]. [Accessed: 18 Sep 2019]. Dutch. Available from: https://www.ggdreisvaccinaties.nl/tarieven

[34] Vaccinatiecentrum. Prijzen vaccinaties en medicatie. [Tropical health advice and vaccination. Prices of vaccines and medications]. [Accessed: 18 Sep 2019]. Dutch. Available from: https://vaccinatiecentrum.nl/index.php/vaccinaties-en-medicatie/prijzen

