# Supplementary material for: Rabies vaccination strategies in the Netherlands in 2018: a cost evaluation
Source: Euro Surveill. 2020 Sep 24;25(38):1900716. doi: 10.2807/1560-7917.ES.2020.25.38.1900716 (PMC7533619; doi:10.2807/1560-7917.ES.2020.25.38.1900716)
Supplement: Supplement [file 19-00716_SUIJKERBUIJK_Supplement.pdf]

Supplementary material: Rabies vaccination strategies in the Netherlands in 2018: a cost evaluation

"This supplementary material is hosted by Eurosurveillance as supporting information alongside the article Rabies vaccination strategies in the Netherlands in 2018: a cost evaluation, on behalf of the authors, who remain responsible for the accuracy and appropriateness of the content. The same standards for ethics, copyright, attributions and permissions as for the article apply. Supplements are not edited by Eurosurveillance and the journal is not responsible for the maintenance of any links or email addresses provided therein."

Additional information on pre-travel visitors

More specifically, we used the number of travellers to rabies endemic countries and the number of vaccinated individuals visiting the MHS travel clinic stratified by age and sex. We extrapolated these data to the whole country assuming that data from MHS Hart voor Brabant and MHS Amsterdam respectively represented the less and highly urbanized regions. In total, around 300,000 persons were assumed to visit the MHS prior to their travel to a rabies-endemic country of which ca 10% would be vaccinated. Data from Statistics Netherlands were used to divide the Dutch population into living in an area with a low and high degree of urbanization [1, 2]. We assumed that everyone who receives rabies vaccine will be fully vaccinated because in the Netherlands a vaccination series is only started if there is enough time and opportunity to complete it before travelling in order to be sufficiently protected during travel. The number of travellers consulting a GP or other type of travel clinic (non-MHS) before travel were based on data from a case-control study of patients who may have been exposed to rabies [3]. As data were lacking, we assumed the same distribution in age and sex of travellers to rabies endemic countries and travel destinations for travellers consulting a GP as for the travellers visiting the MHSs. For those travellers consulting a GP a mean vaccination uptake for all ages was based on the vaccination uptake among travellers visiting MHS travel clinics. The number of pre-travel visitors and travel destinations are presented in table S1 and S2 in the appendix file.

Additional information on Eurocross Assistance (ECA)

ECA (Leiden, the Netherlands) is one of the leading medical assistance organizations in the Netherlands, covering approximately 30% of the Dutch health care and travel insurance market. On behalf of several insurance companies ECA assists Dutch citizens who have encountered medical problems abroad. The ECA database includes information provided by the patients themselves, concerning injuries or other medical problems. Travellers who encountered an AAI actively contact ECA for medical advice, which is given according to the WHO recommendations. During these notifications, additional questions such as PrEP status and exposure category were asked in order to be able to give proper advice.

Table S1. Travelers to rabies endemic countries consulting MHSs, GPs and other clinics between July 1st 2016 and July 1st 2017

	Visitors to rabies endemic country (whereof vaccinated (number and in% of visitors))	0-15 years	16-30 years	31-45 years	45-60 years	>60 years %
		% visitors (% vaccinated)				
**MHS Amsterdam**	20,851 (2424, 11.6%)	11.06 (3.47)	44.71 (15.82)	24.70 (11.46)	12.93 (7.20)	6.59 (6.19)
**MHS Hart voor Brabant**	14,828 (1305, 8.8%)	9.30 (11.80)	40.50 (15.13)	17.20 (6.67)	22.40 (4.60)	10.60 (9.07)
**All MHSs extrapolated to NL**	300,630 (32,212, 10.7%)	10.42 (7.22)	43.18 (15,51)	21.98 (9,30)	16.36 (6,03)	8.04 (7,49)
**All GPs**	32,820	10.42 (10.57)	43.18 (10.57)	21.98 (10.57)	16.36 (10.57)	8.04(10.57)
**Other**	19,692	10.42(10.57)	43.18 (10.57)	21.98 (10.57)	16.36 (10.57)	8.04 (10.57)
**Total**	353,141					

MHS=Municipal Health Service, GP=General Practitioner

Table S2. Travel destinations of travellers to rabies endemic countries between July 1st 2016 and July 1st 2017

	South-eastern Asia	Western Asia	Southern Asia	Eastern Asia	Caribbean	Central America	Southern America	Southern Africa	Western Africa	Middle Africa	Eastern Africa	Northern Africa	Southern Europe	Eastern Europe	Total
**MHS Amsterdam**	47.00%	5.52%	12.57%	2.89%	2.15%	3.72%	9.62%	5.21%	1.87%	0.23%	6.75%	2.29%	0.00%	0.09%	100%
**MHS Hart voor Brabant**	50.40%	3.58%	11.76%	2.63%	2.66%	5.82%	4.57%	9.85%	3.96%	0.00%	5.86%	1.57%	1.89%	0.00%	100%

MHS=Municipal Health Service

Table S3A. Number of vaccinated persons, changes in AAI and annual costs of PrEP and PEP following a 1.5 increase of vaccination uptake in risk groups

	Reference value (new guidelines IM)	Travellers < 35	Difference with reference value	Travellers to south Eastern and Western Asia	Difference with reference value	Men	Difference with reference value
**Nr of visitors vaccination center prior to travel to rabies endemic country**	353,141	353,141	0	353,141	0	353,141	0
**Nr of vaccinated persons**	37,317	49,284	11,967	47,116	9799	45,884	8567
**Nr of AAI in vaccinated travellers***	322	439	117	428	106	397	75
**Nr of AAI in unvaccinated travellers not needing RIG****	665	593	-72	600	-65	619	-46
**Nr of AAI in unvaccinated travellers needing RIG*****	413	368	-45	372	-41	384	-29
**Costs before travel (€)**							
**Consultation**	6,807,812	6,934,611	126,798	6,911,643	103,831	6,898,587	90,775
**Vaccination**	3,927,198	5,186,605	1,259,407	4,958,480	1,031,282	4,828,810	901,611
**TOTAL (before travel)**	10,735,010	12,121,215	1,386,205	11,870,123	1,135,113	11,727,397	992,387
**Costs after potential exposure (€)**							
**Vaccinations and RIG**	1,086,366	984,408	-101,948	993,621	-92,745	1,021,260	-65,106
**Consultation and coordination**	480,948	444,655	-36,293	447,935	-33,014	457,773	-23,175
**Repatriation**	64,784	51,090	-13,694	50,749	-14,035	52,688	-12,096
**Lost holidays**	422,982	406,665	-16,316	406,260	-16,722	408,569	-14,412
**Evacuation**	56,397	44,475	-11,921	44,179	-12,218	45,866	-10,530
**TOTAL (after potential exposure) (€)**	2,111,476	1,931,293	-180,183	1,942,744	-168,732	1,986,157	-125,319
**GRAND TOTAL (€)**	**12,846,486**	**14,052,509**	**1,206,022**	**13,812,867**	**966,380**	**13,713,554**	**867,067**
**Cost-effectiveness ratio (CER)**							
**Incremental costs (€) per additional vaccinated person**			101		99		101
**Incremental costs (€) per RIG administration avoided**			26,970		23,760		30,370

* both category II and category III injuries, ** category II injuries, *** category III injuries, PrEP= pre-exposure prophylaxis, PEP=post-exposure prophylaxis, RIG= rabies immunoglobulin, AAI=animal associated injury, IM = intramuscular administration, ID = intradermal administration

S3B. Number of vaccinated persons, changes in AAI and annual costs of PrEP and PEP following a 1.5 increase of vaccination uptake in risk groups

	Reference value (new guidelines IM)	Travellers to south America	Difference with reference value	Travellers having at least one risk factor	Difference with reference value	Travellers having all risk factors together	Difference with reference value
**Nr of visitors vaccination center prior to travel to rabies endemic country**	353141	353141	0	353141	0	353141	0
**Nr of vaccinated persons**	37317	39112	1795	54605	17288	40731	3414
**Nr of AAI in vaccinated travellers***	322	333	11	479	157	363	41
**Nr of AAI in unvaccinated travellers not needing RIG****	665	658	-7	568	-97	640	-25
**Nr of AAI in unvaccinated travellers needing RIG*****	413	409	-4	353	-60	397	-16
**Costs before travel (€)**							
**Consultation**	6,807,812	6.826.831	19.018	6,990,992	183,180	6,843,991	36,179
**Vaccination**	3,927,198	4.116.096	188.898	5,746,606	1,819,408	4,286,537	359,338
**TOTAL (before travel)**	10,735,010	10.942.927	207.917	12,737,598	2,002,588	11,130,527	395,517
**Costs after potential exposure (€)**							
**Vaccinations and RIG**	1,086,366	1,077,152	-9,213	949,398	-136,968	1,050,742	-35,624
**Consultation and coordination**	480,948	477,669	-3,279	432,193	-48,755	468,267	-12,681
**Repatriation**	64,784	62.644	-2.140	44,602	-20,182	60,181	-4,603
**Lost holidays**	422,982	420.433	-2.549	398,935	-24,047	417,498	-5,484
**Evacuation**	56,397	54.534	-1.863	38,827	-17,569	52,390	-4,007
**TOTAL (after potential exposure) (€)**	2,111,476	2,092,432	-19,044	1,863,955	-247,522	2,049,078	-62,398
GRAND TOTAL (€)	12,846,486	13,035,359	188,873	14,601,553	1,755,522	13,179,606	333,119
**Cost-effectiveness ratio (CER)**							
**Incremental costs (€) per additional vaccinated person**			105		102		98
**Incremental costs (€) per RIG administration avoided**			46,750		29,220		21,320

* both category II and category III injuries, ** category II injuries, *** category III injuries, PrEP= pre-exposure prophylaxis, PEP=post-exposure prophylaxis, RIG= rabies immunoglobulin, AAI=animal associated injury, IM = intramuscular administration, ID = intradermal administration

Figure S1 and Figure S2 show that the values that are related to the extra costs and vaccinations, respectively RIG avoided, are on a virtual linear line.

Figure S1: number of additional vaccinated persons plotted against the incremental costs

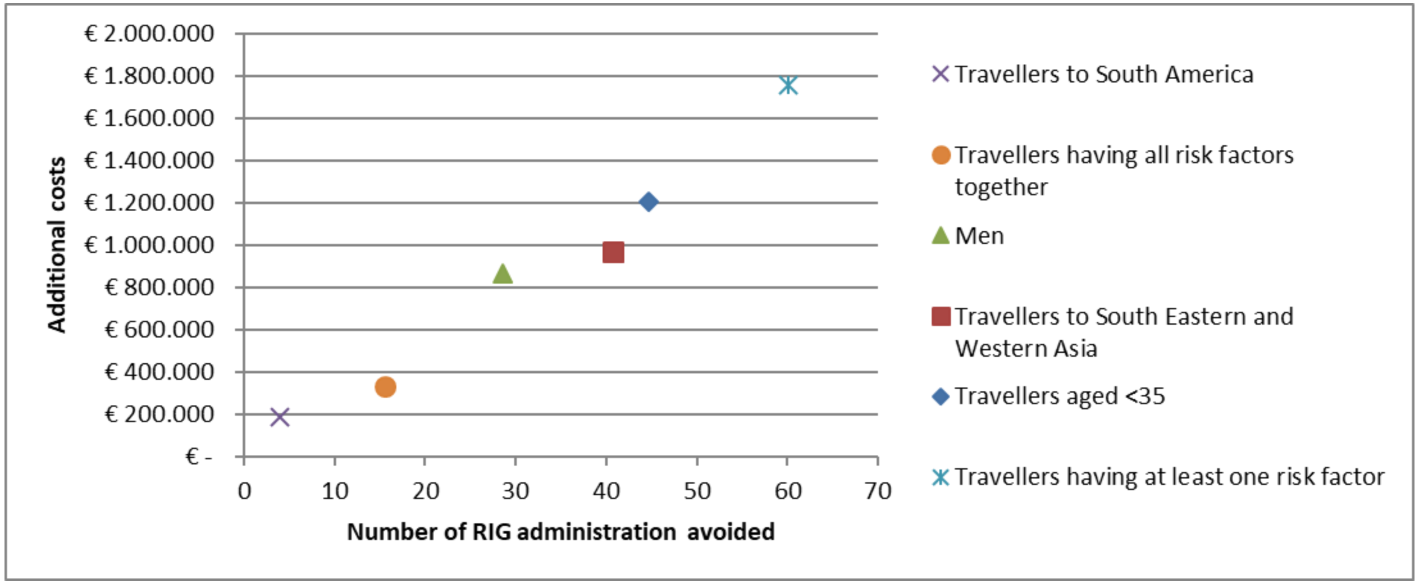

Figure S2: number of RIG administration avoided plotted against the incremental costs

Supplemental information about cost estimations

RIG and vaccine administration

Mean costs for RIG administered abroad were based on ECA data and for RIG administered in the Netherlands were based on data from RIVM. All costs involved in the administration of rabies vaccinations were based on Dutch prices independent of the country of administration. Vaccination costs were retrieved from a website from the Dutch national healthcare institute [4]. The average number of ID applications that could be given to individuals in one session from one vial of 1 ml (an opened vial has to be used within 6-8 hours) was based on practical experience from the LUMC vaccination clinic. GP administration and consultation costs were based on Dutch guidelines for economic evaluations in health care [5].

Consultation costs, management AAI

Consultation costs with respect to PrEP and management costs with respect to PEP at MHSs and outpatient clinics were based on a survey among clinicians and nurses from four MHSs and the LUMC vaccination clinic. The mean time spent with pre-travel advice and on management of PEP was collected and multiplied by the average hourly wage of nurses and clinicians involved.

Repatriation and evacuation

The mean costs of repatriation (to the Netherlands) and evacuation (to another country abroad) both needed for the administration of RIG were obtained from the ECA registry containing information of travellers with an AAI between 2016 and 2018.

Lost holidays

Costs of lost holidays were assumed equal to productivity losses, following the Dutch guideline for social cost-benefit analyses [6]. Lost holidays, due to repatriation and evacuation were retrieved from the case control study among travellers having an AAI [3]. The number of lost holidays were multiplied by the average hourly wage adjusted for the average employment rate in all age groups [5, 7].

Costs before travelling	€
First consultation (per person) at MHS:<br>20 minutes visit with a public health nurse (€51/hour); in 7.7% of all cases a clinician is involved (€76/hour) during 6 minutes. Total costs are corrected as on average 1.4 persons visit the clinic per consultation.	13.10
Second consultation (per person) at MHS:<br>9 minutes visit with a public health nurse (€51/hour); in 6.3% of all cases a clinician is involved (€76/hour) during 1 minute. Total costs are corrected as on average 1.4 persons visit the clinic per consultation.	5.45
Vaccine costs per ID administration:<br>7.6 doses per vial (€51.64)	6.81
**Costs after AAI**	**€**
Management MHS for persons needing RIG:<br>190 minutes with a public health nurse (€51/hour) and 90 minutes for a clinician (€76/hour)	276
Management RIVM for persons needing RIG:<br>National Coordination Centre for Communicable Disease Control:<br>3 minutes for secretary (€81/hour), 20 minutes for adviser (€110/hour) and 15 minutes for clinician (€132/hour)<br>Department for Vaccine Supply:<br>2 hours for adviser (€94/hour) and 1 hour for manager (€110/hour)	371
Administration RIG:<br>50% of all RIG is administered at the MHS at a cost of €17.70; 40% is administered at a GP at a cost of €33.80 and 10% is administered at the ED at a cost of €93.10	31.76
Management MHS for persons not needing RIG:<br>77 minutes visit with a public health nurse (€51/hour) and 26 minutes for clinician (€76/hour)	98.64
Administration vaccination first visit:<br>9 minutes visit with a public health nurse (€51/hour) in 6.3% of all cases a clinician is involved (€76/hour) during 1 minute.	7.63

References

[1] CBS. Trends in Nederland 2017. [cited 2018; Available from: https://longreads.cbs.nl/trends17/maatschappij/cijfers/bevolking/

[2] CBS. Nederlanders in stedelijke gebieden. 2018 [cited 2018; Available from: https://www.cbs.nl/nl-nl/nieuws/2005/08/meer-inwoners-in-stedelijke-omgeving

[3] Bantjes S, Haverkate M, Ruijs H, et al. Predictors of possible rabies exposure in travelers: a case-control study. European Journal of Public Health 2018;28(suppl_4).

[4] Nederlands-Zorginstituut. [cited; Available from: www.medicijnkosten.nl

[5] Zorginstituut_Nederland. Richtlijn voor het uitvoeren van economische evaluaties in de gezondheidszorg. Amsterdam: Zorginstituut Nederland; 2015.

[6] Koopmans C, Heyma A, Hof B, et al. Werkwijzer voor kosten-batenanalyse in het sociale domein. Amsterdam: SEO Economisch Onderzoek; 2016.

[7] Statistics_Netherlands. Employment rate. 2019 [cited; Available from: https://opendata.cbs.nl/statline/#/CBS/nl/dataset/82647NED/table?ts=1544012763349
